# Comparison Study of Extraction Accuracy of 3D Facial Anatomical Landmarks Based on Non-Rigid Registration of Face Template

**DOI:** 10.3390/diagnostics13061086

**Published:** 2023-03-13

**Authors:** Aonan Wen, Yujia Zhu, Ning Xiao, Zixiang Gao, Yun Zhang, Yong Wang, Shengjin Wang, Yijiao Zhao

**Affiliations:** 1 Center of Digital Dentistry, Peking University School and Hospital of Stomatology & National Center of Stomatology & National Clinical Research Center for Oral Diseases & National Engineering Research Center of Oral Biomaterials and Digital Medical Devices & Beijing Key Laboratory of Digital Stomatology & NHC Research Center of Engineering and Technology for Computerized Dentistry, Beijing 100081, China; 2 Institute of Medical Technology, Peking University Health Science Center, Beijing 100191, China; 3 Hospital of Stomatology Lanzhou University, Lanzhou 730031, China; 4 Department of Electronic Engineering, Tsinghua University, Beijing 100084, China

**Keywords:** 3D face template, anatomical landmarks, non-rigid registration, non-rigid deformation

## Abstract

(1) Background: Three-dimensional (3D) facial anatomical landmarks are the premise and foundation of facial morphology analysis. At present, there is no ideal automatic determination method for 3D facial anatomical landmarks. This research aims to realize the automatic determination of 3D facial anatomical landmarks based on the non-rigid registration algorithm developed by our research team and to evaluate its landmark localization accuracy. (2) Methods: A 3D facial scanner, Face Scan, was used to collect 3D facial data of 20 adult males without significant facial deformities. Using the radial basis function optimized non-rigid registration algorithm, TH-OCR, developed by our research team (experimental group: TH group) and the non-rigid registration algorithm, MeshMonk (control group: MM group), a 3D face template constructed in our previous research was deformed and registered to each participant’s data. The automatic determination of 3D facial anatomical landmarks was realized according to the index of 32 facial anatomical landmarks determined on the 3D face template. Considering these 32 facial anatomical landmarks manually selected by experts on the 3D facial data as the gold standard, the distance between the automatically determined and the corresponding manually selected facial anatomical landmarks was calculated as the “landmark localization error” to evaluate the effect and feasibility of the automatic determination method (template method). (3) Results: The mean landmark localization error of all facial anatomical landmarks in the TH and MM groups was 2.34 ± 1.76 mm and 2.16 ± 1.97 mm, respectively. The automatic determination of the anatomical landmarks in the middle face was better than that in the upper and lower face in both groups. Further, the automatic determination of anatomical landmarks in the center of the face was better than in the marginal part. (4) Conclusions: In this study, the automatic determination of 3D facial anatomical landmarks was realized based on non-rigid registration algorithms. There is no significant difference in the automatic landmark localization accuracy between the TH-OCR algorithm and the MeshMonk algorithm, and both can meet the needs of oral clinical applications to a certain extent.

## 1. Introduction

The anatomical and morphological features of the human face are often considered facial anatomical landmarks during the diagnosis and treatment of oral craniomaxillofacial diseases [1]. The clinical diagnosis, treatment planning, and evaluation of treatment outcomes for oral and maxillofacial surgery, orthodontic, and prosthodontic patients are often based on the morphological analysis of facial anatomical landmarks [2,3,4,5,6,7,8,9,10,11]. The traditional methods mostly employ anatomical landmarks on the two-dimensional (2D) image of the patient’s face for measurement. There are also reports of directly marking the anatomical landmarks on the patient’s face for measurement [12]. However, 2D image measurement lacks the depth information of 3D facial morphology, and directly marking landmarks on the face has the potential risk of facial trauma. With the development of three-dimensional (3D) optical non-invasive scanning technology, the application of facial 3D morphological data has increasingly gained clinical attention [13]. Automatic, accurate, and rapid identification of facial anatomical landmarks based on 3D facial data is currently a hot topic of research.

The existing automatic algorithms to determine anatomical landmarks using 3D facial data mainly include geometric feature algorithms [14,15,16,17,18] and artificial intelligence algorithms [19,20,21,22,23,24]. Geometric feature algorithms help determine facial anatomical landmarks by analyzing the changes in facial curvature characteristics and combining the prior knowledge of facial geometrical morphology. This method is suitable when there are significant changes in facial morphological features, and therefore, the number of anatomical landmarks that can be automatically determined is limited. The artificial intelligence algorithm uses training set data for deep learning to automatically determine facial anatomical landmarks. Based on the training set data with different annotation information, the corresponding number of anatomical landmarks can be automatically determined. The number and location of anatomical landmarks that can be determined by each algorithm model lack flexibility, and improvements are necessary for clinical universality.

Our research team had previously reported a method to automatically determine facial anatomical landmarks by combining 3D face templates and the non-rigid registration algorithm MeshMonk [25] (referred to as “template method”). We preliminarily tested the effect and feasibility of the “template method” using the 3D facial data of five individuals with no significant facial deformity and that of five with mild mandibular deviation [26]. Compared with geometric feature algorithms and artificial intelligence algorithms, the number of 3D facial anatomical landmarks that can be automatically determined by the template method is not limited by facial anatomical features and has good flexibility. Therefore, the template method has good application potential and clinical suitability in an oral clinic. However, in our previous study, we did not conduct an in-depth evaluation of the landmark localization accuracy of the template method.

Therefore, our research objectives are as follows: (1) Based on the non-rigid registration algorithm (TH-OCR) developed by our research team, realize automatic determination of 3D facial anatomical landmarks; (2) compare and analyze the automatic landmark localization accuracy of TH-OCR and MeshMonk, and provide corresponding reference for the application of the template method in an oral clinic.

## 2. Materials and Methods

### 2.1. Subjects

Twenty adult males who came to Peking University School and Hospital of Stomatology were recruited. The inclusion criteria were as follows: (1) The facial morphology was normal without obvious facial deformities. (2) No facial defects, traumas, no obvious facial asymmetry. The exclusion criteria were as follows: (1) After oral clinical diagnosis, suffering from facial deformities, traumas, defects, etc. (2) Patients who do not accept or are not comfortable with optical scanning of the face. This study was approved by the Bioethics Committee of Peking University Hospital of Stomatology (PKUSSIRB-202164079). The purpose and procedures of this study were fully explained to all subjects, and written informed consent was obtained before participation.

### 2.2. Experimental Equipment and Software

Face Scan (3D-Shape Corp, Erlangen, Germany), a 3D optical sensor, was used to collect the 3D facial data of the participants, using the following parameters: scanning speed, 0.8 s; scanning accuracy, 0.2 mm; scanning angle, 270°–320°; the imaging principle was raster scanning using 5 million charge-coupled device pixels, with approximately 10,000 data points and 20,000 triangular meshes.

The reverse engineering software Geomagic Studio 2013 (3D Systems, Morrisville, NC, USA) was used to preprocess each participant’s 3D facial data and manually select the anatomical landmarks. The Procrustes analysis (PA) algorithm in MATLAB R2019b (MathWorks, Natick, MA, USA) was used to calculate the scaling factor of the 3D face template. Meshlab 2020 (Open source, Tuscany, Italy) was used for data preparation before applying the non-rigid registration procedures. The non-rigid registration algorithm MeshMonk was used for deformation registration between the 3D face template of the control group (MM group) and the 3D facial data of the participants. The non-rigid registration algorithm TH-OCR developed by our research team was used for deformation registration between the 3D face template of the experimental group (TH group) and 3D facial data of the participants.

The algorithm MeshMonk runs in MATLAB R2019b, and TH-OCR runs in python 3.8. The hardware configuration for the algorithm to run was Intel Xeon Silver 4210R, 2.40 GHz GPU, and 192 GB of RAM.

### 2.3. Three-Dimensional Facial Data Collection and Processing

Face Scan was used to collect 3D facial data of adult males without significant facial deformities. Instrument calibration was performed before scanning to ensure accurate imaging. In accordance with the investigators’ instructions, the participants sat 135 cm in front of the instrument with a natural head position while looking straight ahead with both eyes and maintaining the Frankfort plane parallel to the ground. The participants’ face was completely exposed up to the hairline and until the ears on the left and right, without glasses or hair covering the face. Their facial expressions were naturally relaxed. After scanning, the 3D facial data was saved in the OBJ format.

In the reverse engineering software Geomagic Studio 2013, the 3D facial data of the participants were processed. Redundant data were deleted, and the range of retained data included that up to the hairline, left and right to the tragus, bypassing the mandibular angle, and along the mandible to the submental chin, with repair of the defect area on the facial margin. The spatial pose of the 3D facial data was adjusted in the software to ensure that the mid-sagittal plane was parallel to the YZ plane to achieve a natural head position. The data were saved as OBJ files (FaceModel_Patient).

A single operator with clinical experience, who was also skilled in the operation of Geomagic Studio 2013 software, selected 32 facial anatomical landmarks routinely used in dental clinics, including the trichion, glabella, and pronasale, on the 3D facial data of each participant. These included 10 midline and 22 bilateral points. The number, name, and abbreviation of these anatomical landmarks are presented in Table 1. Three consecutive selections were made, and the average coordinate value of the landmarks was considered the reference value for manual selection (Point_Ref).

### 2.4. Determination of 3D Facial Anatomical Landmarks

**Figure 1:**
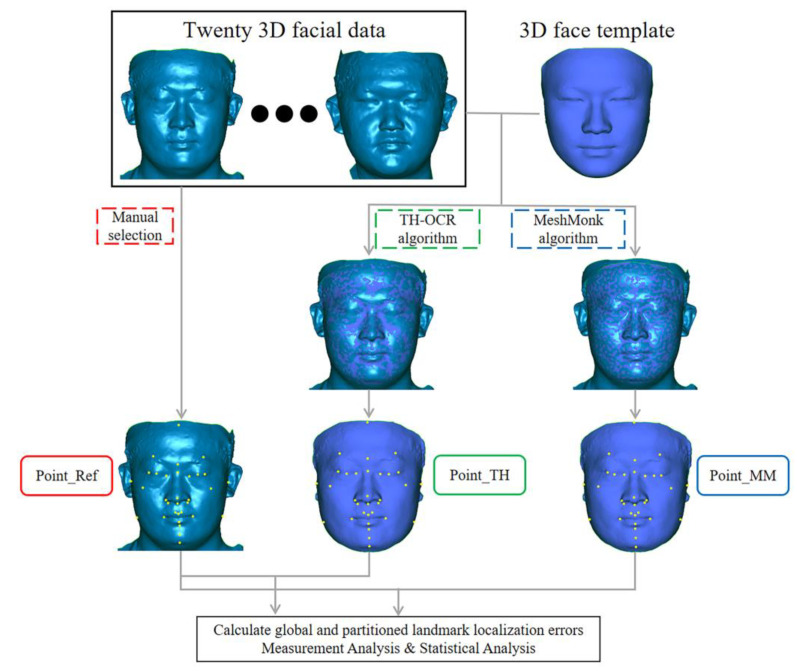
Flow chart depicting the experiment method.

A flow chart depicting the experiment method is shown in Figure 1.

**Figure 2:**
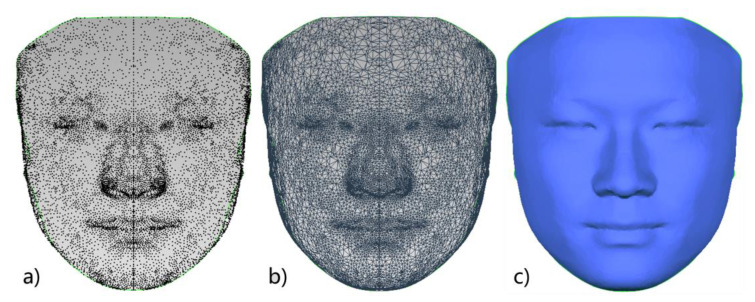
Three-dimensional face template: (**a**) 3D face template in point cloud form; (**b**) 3D face template in triangular mesh form; (**c**) 3D face template in smooth surface form.

**Figure 3:**
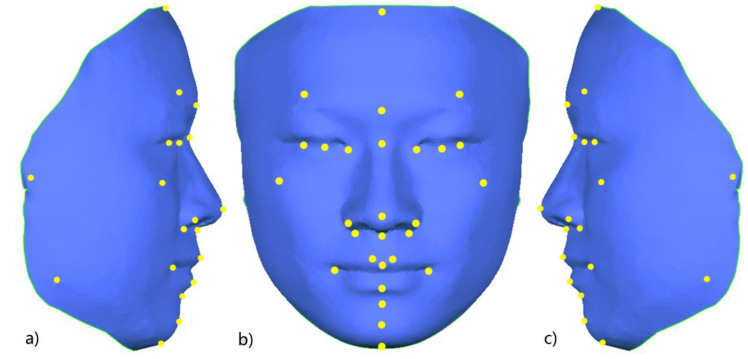
Schematic diagram of 32 facial anatomical landmarks on the 3D face template; (**a**) right lateral view; (**b**) frontal view; (**c**) left lateral view.

In a previous study, our research team had constructed a 3D face template (FaceModel_Mask) based on the average 3D facial data of 30 Chinese adult males with good facial symmetry, as shown in Figure 2. This 3D face template has 19,534 triangular faces and 9856 vertices, of which 216 vertices are on the midline of the face, and the X coordinate of all midline points is zero. There are 4820 vertices on the left and right sides, and the vertices on both sides are symmetric based on the midline of the face and have a one-to-one correspondence [26]. Compared with the 3D face templates constructed in previous related studies [25,27,28], the 3D face template in this study has the Chinese 3D facial anatomical features. Its data range covers the whole face, including the positions of facial anatomical landmarks commonly used in oral clinical practice, which provides the necessary data basis for this study. Thirty-two facial anatomical landmarks shown in Table 1 were selected from the 9856 vertices of the 3D face template, as shown in Figure 3. The vertex indices were recorded, as shown in Table 1.

Based on the above-mentioned 3D face template and the open-source non-rigid registration algorithm (MeshMonk), we proposed a “template method” to automatically determine the 3D facial anatomical landmarks. The principle of the template method was as follows: MeshMonk was used to deform and register the 3D face template to the target 3D facial data. The vertex indexes before and after the deformation of the 3D face template remained unchanged. Therefore, based on the recorded vertex indexes, the 3D coordinates of the corresponding vertices on the deformed 3D face template can be automatically obtained. In this way, the effect of automatically determining the anatomical landmarks of the target 3D facial data was realized.

In this study, based on the process of the template method, the non-rigid registration algorithm (TH-OCR) developed by our research team was used to realize the automatic determination of the 3D facial anatomical landmarks. Taking TH-OCR as the experimental group and MeshMonk as the control group, the automatic landmark localization accuracy of the two algorithms was analyzed and evaluated. The automatic landmark localization steps of the TH-OCR algorithm were as follows (the data of one participant were used to illustrate the process):

Step 1: Using the Meshlab 2020 software, a total of 8 facial anatomical landmarks, including the bilateral tragion, endocanthion, pronasale, cheilion, and gnathion, were selected on the 3D face template (FaceModel_Mask). The 3D coordinate data of the landmarks was set as .pp and .csv files. Similarly, the above 8 anatomical landmarks were selected on the participant’s 3D facial data (FaceModel_Patient), and the 3D coordinate data of the landmarks was again saved as .pp and .csv files. The above 8 facial anatomical landmarks were used for the initialization process of non-rigid registration.

Step 2: The landmark set coordinate data (.csv format) of FaceModel_Mask and FaceModel_Patient was imported in MATLAB R2019b software. The Procrustes analysis algorithm was used to calculate the overall scaling factor for FaceModel_Mask and to save the scaling factor in .txt format.

Step 3: The FaceModel_Patient, FaceModel_Mask, their landmark set data (.pp format), and the overall scaling factor of FaceModel_Mask were imported into the TH-OCR algorithm. The algorithm first performed rigid registration based on the 8 landmarks of the two models and unified FaceModel_Patient and FaceModel_Mask to the same spatial scale based on the overall scaling factor of FaceModel_Mask. Then, based on the radial basis function, according to the corresponding relationship between the two sets of landmark data, FaceModel_Mask was elastically deformed, and the 3D shape of FaceModel_Mask was initially close to that of FaceModel_Patient. Then, based on the non-rigid ICP algorithm, FaceModel_Mask was registered to FaceModel_Patient, so that it was further approximated to the 3D shape of FaceModel_Patient. Based on the pre-determined 32 anatomical landmark indexes on FaceModel_Mask, the coordinates of these anatomical landmarks on the deformed FaceModel_Mask were obtained. This resulted in the automatic determination of 3D facial anatomical landmarks by the TH-OCR algorithm.

**Figure 4:**
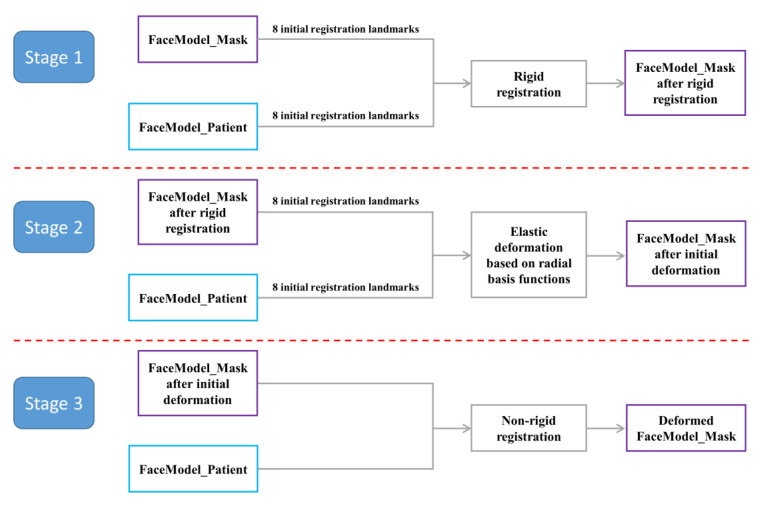
Flow chart of the TH-OCR algorithm.

The algorithm flow in step 3 is shown in Figure 4. Part of the function formula involved in step 3 was as follows:

Rigid registration part:

(1) $$F\left(\begin{array}{c} r\\ q \end{array}\right)=\frac{1}{K}\overset{K}{\underset{j=1}{\sum }}\|f_{j}^{t}-R\left(q_{R}\right)f_{j}^{s}-q_{T}\|{}^{2}.$$

where $\left\{f_{j}^{t}|j\in \left[1,K\right]\right\}$ is the set of landmarks of FaceModel_Patient; $\left\{f_{j}^{s}|j\in \left[1,K\right]\right\}$ is the set of landmarks of FaceModel_Mask; $q_{R}$ stands for rotation matrix; $q_{T}$ stands for translation vector; K is the number of landmarks. Rigid registration of FaceModel_Patient and FaceModel_Mask was realized based on this function formula.

Preliminary elastic deformation based on radial basis function was as follows:

(2) $$p_{i}^{new\;}=F\left(p_{i}\right)=\overset{K}{\underset{j=1}{\sum }}\alpha_{j}\phi \left(p_{i}-f_{j}^{s}\right)+a+bx_{i}+cy_{i},i\in \left[1,N\right].$$

where $p_{i}^{\;}$ = (*x_i_,y_i_,z_i_*) is the ith vertex of FaceModel_Mask; $p_{i}^{new\;}$ is the ith vertex of FaceModel_Mask after the initial elastic deformation; N is the number of vertices of FaceModel_Mask; $\alpha_{j}$ is the deformation parameter; a,b,c is the affine transformation parameter; $\phi \left(r\right)=\exp(-k\|r\|)$ is the radial basis function, where k is a positive parameter. The preliminary elastic deformation of FaceModel_Mask was realized based on the correspondence between the landmark sets of FaceModel_Patient and FaceModel_Mask and this function formula.

Non-rigid registration part. Objective function for the non-rigid registration procedure was as follows:

(3) $$E=\alpha E_{d}+\beta E_{s}+\gamma E_{f}.$$

where $E_{d}$ is the data term error; $E_{s}$ is the smoothing term error; and $E_{f}$ is the feature point registration error; α,β,γ is the weighting parameter.

$E_{d}$ was defined as follows:

(4) $$E_{d}=\overset{N}{\underset{i=1}{\sum }}w_{i}\|X_{i}p_{i}^{new\;}-D_{i}\|{}^{2}.$$

where $X_{i}$ represents the affine transformation matrix; $D_{i}$ is the closest vertex on FaceModel_Patient to $p_{i}^{new\;}$; $w_{i}$ is the weight. If a corresponding point for $p_{i}^{new\;}$ could not be found on FaceModel_Patient, $w_{i}$ was set to 0, otherwise it was set to 1.$\left\|\cdot \right\|$ takes the 2 norm.

$E_{s}$ was defined as follows:

(5) $$E_{s}=\underset{\left\{\left(p_{i}^{new\;},p_{j}^{new\;}\right)\in edge(p)\right\}}{\sum }||X_{i}-X_{j}||{}^{2}.$$

where $\left(p_{i}^{new\;},p_{j}^{new\;}\right)$ is the line segment connecting $p_{i}^{new\;}$ and $p_{j}^{new\;}$; edge(p) is the set of all edges in the FaceModel_Mask after the initial elastic deformation.

$E_{f}$ was defined as follows:

(6) $$E_{f}=\overset{m}{\underset{l=1}{\sum }}\|X_{K_{l}}p_{K_{l}}-D_{K_{l}}\|{}^{2}.$$

where $K_{l}\left(l=1,2,\ldots ,m\right)$ stands for landmarks.

The process of non-rigid registration was as follows:

1. Initialize *X_i_^0^*(*i = 1*, *…*, *N*)*, k = 0*;
2. Based on a fixed set of α,β,γ parameters (in this study, *α* = 1, the iteration parameters of *β* were: 20, 10, 10, 10, 5, 1, and the iteration parameters of *γ* were: 100, 100, 20, 5, 0.05, 0.005),
  ①. For each vertex $p_{i}^{new\;}$*^k^* on FaceModel_Mask, find the closest vertex on FaceModel_Patient as the corresponding point $D_{i}$*^k^*;
  ②. Calculate ***X**_i_^k^* (*i =* 1, …, *N*) to minimize error *E*;
  ③. Update FaceModel_Mask, the new FaceModel_Mask = {$p_{i}^{new\;}$*^k+1^|i = 1*, …, *N*} = {*X_i_^k^*$p_{i}^{new\;}$*^k^| i =* 1, *…*, *N*};
  ④. Repeat ①–③ until $\sum_{i=1}^{N}||X_{i}^{k}-X_{i}^{k+1}||<\varepsilon ,k=k+1$;
3. Repeat step 2 after changing the parameters. Output the deformed FaceModel_Mask.

The above operation was repeated, and the TH-OCR algorithm was used to complete the determination of anatomical landmarks on the 3D facial data of the 20 participants. The coordinate values of 32 anatomical landmarks for each participant’s data were recorded as the experimental group of this study (Point_TH).

Based on the above data, the non-rigid registration algorithm MeshMonk was used in the control group to achieve the registration of FaceModel_Patient and FaceModel_Mask. The determination of anatomical landmarks using the 3D facial data of 20 participants was completed, and the coordinate values of 32 anatomical landmarks for each participant’s data were recorded as the control group (Point_MM).

### 2.5. Measurement Analysis

For the 3D facial data of 20 adult males without significant facial deformities included in this study, 32 facial anatomical landmarks manually determined by experts were used as reference values (Point_Ref). The Euclidean distance between the coordinate values of the 32 facial anatomical landmarks determined in the Point_TH group, Point_MM group, and Point_Ref group were calculated for each participant and defined as the “landmark localization error”. The mean and standard deviation of the landmark localization error of the 32 facial anatomical landmarks in the Point_TH group and the Point_MM group were calculated (average of 20 patients’ eponymous landmarks). A quantitative analysis of the effect of the two algorithms in determining facial anatomical landmarks was performed.

The face is divided into three parts based on the horizontal plane passing through the eyebrow point and the nasal base point: the upper face, middle face, and lower face. There are 4 anatomical landmarks in the upper face, 17 in the middle face, and 11 in the lower face. The mean and standard deviation of the landmark localization error of the anatomical landmarks in the three face regions was calculated for each participant. In addition, the facial area comprising the trichion, bilateral tragion, bilateral gonion, and gnathion was defined as the facial marginal area in this study, and the area comprising the remaining 26 landmarks was defined as the facial central area. The mean and standard deviation of the landmark localization error of the anatomical landmarks in the marginal and central areas of the face was calculated to investigate the effect of two algorithms on the determination of anatomical landmarks in different regions of the face.

### 2.6. Statistical Analysis

To investigate the consistency and reproducibility of manually selected facial anatomical landmarks by the same operator, the intra-class coefficient (ICC) was calculated.

Using SPSS 21.0 software, the S-W normality test was performed on the mean values of the landmark localization error of the 32 facial anatomical landmarks in the Point_TH and Point_MM groups. The Friedman rank sum test was used to make statistical inferences on the effect of determination of facial anatomical landmarks by the two algorithms. The test level α of 0.05 indicated a statistically significant difference. We analyzed whether there was statistical difference between the two methods.

## 3. Results

The ICC of the facial anatomical landmarks manually selected by the same operator were all >0.95 (0.98–1.00), demonstrating high intra-operator reproducibility.

**Figure 5:**
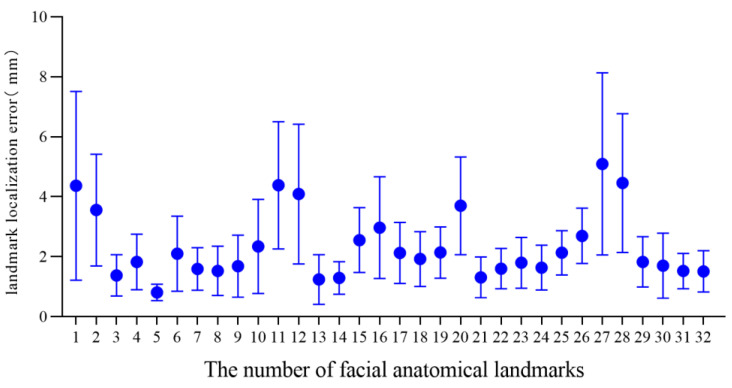
Landmark localization error for each facial anatomical landmark in the TH group.

**Figure 6:**
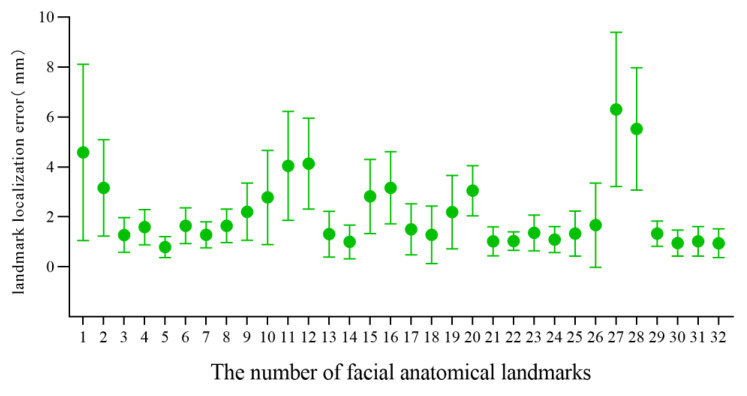
Landmark localization error for each facial anatomical landmark in the MM group.

For the 3D facial data of 20 individuals without significant facial deformity in this study, the mean and standard deviation of the landmark localization error of the 32 facial anatomical landmarks in the TH and MM groups was calculated, as shown in Table 2 and Figure 5 and Figure 6. The average landmark localization error of the 32 landmarks in the Point_TH group was 2.34 ± 1.76 mm; the error for the subnasale was the lowest (0.81 ± 0.27 mm) and that for the right gonion was the greatest (5.10 ± 3.04 mm). The landmark localization error was less than 2 mm for 53.1% landmarks and less than 4 mm for 84.4%. The average landmark localization error of the 32 landmarks in the Point_MM group was 2.16 ± 1.97 mm; the error for the subnasale was the lowest (0.80 ± 0.42 mm) and that for the right gonion was the greatest (6.30 ± 3.09 mm). The landmark localization error was less than 2 mm for 62.5% landmarks and less than 4 mm for 84.4%.

Statistical analysis showed that the S-W normality test *p*-values in the Point_TH and Point_MM group were all less than 0.05, which did not obey the normal distribution. The Friedman rank sum test results showed that there was no significant difference in the landmark localization error of the two algorithms for determining facial anatomical landmarks (*p* > 0.05).

**Figure 7:**
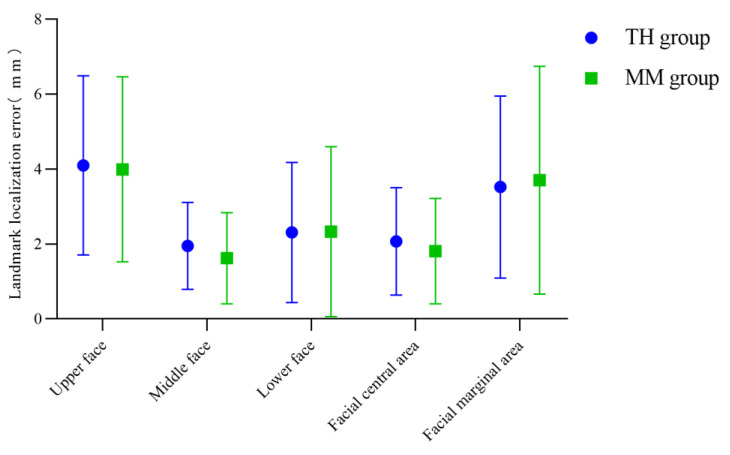
Landmark localization errors in the TH and MM groups based on facial area.

The mean and standard deviation of the landmark localization error of the anatomical landmarks in each facial region of the Point_TH and Point_MM group were calculated, as shown in Table 3 and Figure 7. According to the measurement results, both algorithms are more effective in determining facial anatomical landmarks in the middle face than in the upper and lower face, and the determination of landmarks in the central area of the face was better than that in the marginal area. The determination of landmarks in the central area of the face was slightly better in the Point_MM group than in the Point_TH group, and that in the marginal area was slightly better in the Point_TH group.

## 4. Discussion

### 4.1. Related Studies on Automatic Determination of 3D Facial Anatomical Landmarks

Facial anatomical landmarks play an important role in oral clinical diagnosis and treatment, including preoperative disease diagnosis, treatment planning, or postoperative evaluation of treatment outcomes. Therefore, determination of facial anatomical landmarks has always been a subject of interest. With advances in medical technology, the facial data of oral clinical patients has gradually transitioned from 2D images to 3D digital models, and the determination of facial anatomical landmarks has gradually shifted from 2D to 3D. The traditional method to determine facial anatomical landmarks based on 3D facial data mainly involves manual selection, which requires effort and lacks adequate repeatability and consistency [29,30].

In recent years, algorithm-based methods for determining facial anatomical landmarks have been reported. These algorithms can be broadly classified as geometric feature algorithms and artificial intelligence algorithms, as shown in Table 4. In 2009, Sun et al. [14] proposed a 3D facial landmark determination method based on Shape Index features and geometric constraints, which can automatically determine five facial anatomical landmarks, including the endocanthion and pronasale. The average localization accuracy of this method for facial landmarks is higher than 90%. In 2017, Liang et al. [15] proposed a 3D facial landmark determination method involving HK curvature analysis combined with prior knowledge of facial geometry, which can determine eight facial anatomical landmarks, including the pronasale and cheilion. The method was tested on 3D facial data of neutral expressions, and the average landmark localization error was 4.17 ± 2.53 mm. In 2019, Arpah et al. [17] described the automatic determination of 10 facial anatomical landmarks in the nasolabial region, including the pronasale and cheilion, based on the geometric feature information of 3D facial data, and the average landmark localization error was 2.23 mm. This method is mainly suitable for obvious facial features. The determination of anatomical landmarks, such as the pronasale and cheilion, is good, but that of insignificant facial features is not ideal. A limited number of facial anatomical landmarks can be determined using these methods.

In 2017, Gilani et al. [21] proposed a method to determine facial landmarks based on a Deep landmark identification network. It helped realize the automatic determination of 11 3D facial anatomical landmarks. The FRGC v2.0 face database was used for testing, and the average landmark localization error was 3.0 mm. In 2018, Wang et al. [22] proposed a coarse-to-fine approach to automatically locate the facial landmarks using deep feature fusion on 3D facial geometry data, with an average landmark localization error of 3.96 ± 2.55 mm in determining 14 facial landmarks according to the BU3DFE dataset. In 2019, Wang [19] proposed a method for determining facial landmarks based on denoising auto-encoder networks. This method can determine 22 landmarks on the face, with an average landmark localization error of 3.71 mm. The above-mentioned automatic determination method of facial anatomical landmarks based on an artificial intelligence algorithm is mainly realized through data training. For the training set, face data with a certain amount of anatomical landmark information are manually selected, and the intelligent algorithm trained using it can output the same landmark information as the training set. The number of facial anatomical landmarks that can be determined by such methods is not limited by the 3D morphological features of the face. However, an intelligent algorithm trained by the training set data of a single number of landmarks has limited flexibility. It is mainly reflected in the following: it is impossible to determine the landmarks that were not included during training, and the number of landmarks output by the algorithm is fixed.

### 4.2. Evaluation and Analysis of Automatic Landmark Localization Accuracy of the Template Method

MeshMonk is a non-rigid registration algorithm reported in 2019 [25]. This algorithm can gradually deform and register the 3D face template to the target 3D facial data and make them as similar as possible in 3D shape and spatial position. The number and index of vertices of the 3D face template before and after deformation remain unchanged, but the 3D coordinates of the vertices are changed. Claes P’s research team has used MeshMonk for 3D morphological analysis of the face and disease diagnosis analysis in related research, with an initial attempt to automatically determine 19 anatomical landmarks of the 3D facial data [31,32,33,34]. In this study, a radial basis function optimized non-rigid registration algorithm (TH-OCR) was developed by our research team. The TH-OCR algorithm can deform and register the 3D face template onto the 3D facial data of the subject, and the RMS value of the 3D deviation between the registered template and 3D facial data of the subject was less than 0.2 mm.

In this study, TH-OCR and MeshMonk were used to automatically determine 32 facial anatomical landmarks for 20 samples of three-dimensional facial data without significant facial deformities. The measurement results show that the automatic landmark localization accuracy of TH-OCR and MeshMonk were 2.34 ± 1.76 mm and 2.16 ± 1.97 mm, respectively. The stability of automatic landmark localization accuracy of TH-OCR was slightly better, while the average landmark localization accuracy of MeshMonk was slightly higher. The statistical results showed that there was no statistically significant difference between the automatic landmark localization accuracy of TH-OCR and MeshMonk. By evaluating the automatic landmark localization accuracy of the two algorithms in each area of the face, it was found that the performance of the landmark localization accuracy of the two algorithms was consistent. The landmark localization error in the middle face was the smallest (1.95 ± 1.16 mm; 1.62 ± 1.22 mm), and the landmark localization error in the upper face was the largest (4.10 ± 2.39 mm; 3.99 ± 2.47 mm). As can be seen from Figure 5 and Figure 6, the mean and standard deviation of the landmark localization errors in the TH and MM groups were lower in the nasolabial region. The “template method” is suggested to have good accuracy and robustness in determining the landmarks of significant facial anatomical features (curvature changes significantly), indicating good clinical feasibility. Relevant studies have shown that a landmark localization error within 2 mm is the limit that an operator can achieve [23,35]. For the 32 facial anatomical landmarks in this study, there were 17 and 20 landmarks in the TH and MM groups, respectively, and the landmark localization error was less than 2 mm, which indicated that the “template method” could be applicable in an oral clinic to a certain extent.

Compared with the above-mentioned geometric feature algorithms and artificial intelligence algorithms, the template method has the following advantages: ① According to different needs, the index of the facial anatomical landmarks to be determined can be flexibly recorded on the 3D face template, and the corresponding anatomical landmarks of the target 3D facial data can be determined. Therefore, the number of landmarks that can be automatically determined by the template method is not limited, and the flexibility is good; ② The template method does not require algorithm training and has lower requirements for data and operating environment. Its automatic landmark localization has high efficiency and good accuracy. Therefore, the template method may possibly be applied and popularized in oral clinics.

### 4.3. Advantages and Disadvantages of the TH-OCR Algorithm

The “template method” can help to automatically determine 3D facial anatomical landmarks, including two key parts: 3D face template and non-rigid registration algorithm. In this study, the same 3D face template was used to test the effects of the TH-OCR algorithm developed by our research team and the MeshMonk algorithm to determine 3D facial anatomical landmarks. The measurement results showed that the mean and standard deviation of the landmark localization errors in the TH group (3.52 ± 2.43 mm) were less than those in the MM group (3.70 ± 3.04 mm) when determining landmarks in the facial marginal area (Table 3). This may be because the radial basis function in the TH-OCR algorithm optimizes the non-rigid ICP algorithm. Before deforming and registering the 3D face template to the 3D face data of the individual, the TH-OCR algorithm of our research team preliminarily deforms the 3D face template based on the radial basis function according to the eight manually selected landmarks. The 3D face template is preliminarily close to the 3D shape of the subject’s 3D facial data to improve the speed and accuracy of the subsequent non-rigid ICP algorithm in identifying the corresponding points between the 3D face template and the subject’s 3D facial data. When deforming and registering the 3D face template to the 3D face data of the subject, the TH-OCR algorithm is mainly based on the non-rigid ICP algorithm. In the early stage of deformation registration, the manually selected eight landmarks guide the deformation of the 3D face template. Later, the TH-OCR algorithm controls the data term error, smoothing term error, and feature point registration error of deformation registration by adjusting the weighting parameters of the objective function terms (the constraints on feature point registration errors are from strong to weak, and the constraints on data term errors and smoothing term errors are from weak to strong), and finally completes the deformation registration of the 3D face template. The addition of radial basis functions and the preliminary guidance of manually selected marginal landmarks (bilateral tragion and gnathion) to the deformation of the 3D face template improve the deformation registration effect of the TH-OCR algorithm on the marginal area of the 3D face template to a certain extent. Therefore, the TH-OCR algorithm is slightly superior to the MeshMonk algorithm in determining the landmarks in the facial marginal area. However, the measurement results of this study also showed that when determining the 17 landmarks in the middle area and 26 landmarks in the central area of the face, the mean landmark localization errors in the MM group were 1.62 mm and 1.81 mm, respectively, which were both better than those of the TH group. This reflects that the accuracy of the MeshMonk algorithm in determining the landmarks in the central area of the face is slightly higher than that of the TH-OCR algorithm. The optimization and improvement of the TH-OCR algorithm in this regard requires further research.

### 4.4. Limitations

The “template method” used in this study also has certain limitations in determining facial anatomical landmarks. First, the determination of anatomical landmarks on the edge of the face needs to be improved. Second, this study only evaluated the landmark localization accuracy of the template method on 3D facial data without significant facial deformities. The application effect of the template method on the 3D facial data with different facial deformities needs to be studied further.

## 5. Conclusions

In this study, based on the TH-OCR algorithm developed by our research team, the automatic determination of 3D facial anatomical landmarks was completed. For 3D facial data without significant facial deformities, there was no significant difference between the landmark localization accuracy (2.34 ± 1.76 mm) of the TH-OCR algorithm and the landmark localization accuracy (2.16 ± 1.97 mm) of the MeshMonk algorithm, which can meet the application requirements of an oral clinic to a certain extent. However, the landmark localization accuracy in the facial upper and marginal area of the template method was slightly poor, and it is necessary to optimize the 3D face template and non-rigid registration algorithm. The applicability of the “template method” to patients with different facial deformities needs further study.

## Figures and Tables

**Table 1:** Number, name, abbreviation, and index of facial anatomical landmarks used in this study.

Number	Name	Abbreviation	Index
Midline facial anatomical landmarks			
1	Trichion	Tri	9401
2	Glabella	Gb	9337
3	Nasion	N	7905
4	Pronasale	Prn	9136
5	Subnasale	Sn	7799
6	Labiale superius	Ls	6113
7	Labiale inferius	Li	6039
8	Sublabiale	Sl	4454
9	Pogonion	Pg	3416
10	Gnathion	Gn	2629
Bilateral facial anatomical landmarks (Right/Left)			
11/12	Superciliary ridge	Su	8557/7168
13/14	Endocanthion	En	8246/7542
15/16	Exocanthion	Ex	6818/5455
17/18	Pupil	Pu	8354/7356
19/20	Zygion	Zg	4921/3937
21/22	Alare	Ala	8171/7435
23/24	Subalare	Sal	6387/5673
25/26	Tragion	Tr	545/286
27/28	Gonion	Go	408/346
29/30	Crista philtre	Cph	6222/5929
31/32	Cheilion	Ch	4671/4203

**Table 2:** Landmark localization errors for the 3D facial anatomical landmarks (mm).

Number	Name	TH Group ($\overset{-}{x}$ ± s)	MM Group ($\overset{-}{x}$ ± s)
1	Tri	4.37 ± 3.15	4.58 ± 3.53
2	Gb	3.56 ± 1.86	3.17 ± 1.93
3	N	1.38 ± 0.69	1.28 ± 0.70
4	Prn	1.83 ± 0.93	1.59 ± 0.70
5	Sn	0.81 ± 0.27	0.80 ± 0.42
6	Ls	2.11 ± 1.25	1.65 ± 0.71
7	Li	1.60 ± 0.71	1.28 ± 0.52
8	Sl	1.53 ± 0.82	1.65 ± 0.67
9	Pg	1.69 ± 1.04	2.21 ± 1.15
10	Gn	2.35 ± 1.57	2.78 ± 1.89
11	Su-R	4.38 ± 2.12	4.05 ± 2.18
12	Su-L	4.09 ± 2.33	4.14 ± 1.82
13	En-R	1.24 ± 0.83	1.31 ± 0.91
14	En-L	1.30 ± 0.54	1.00 ± 0.68
15	Ex-R	2.56 ± 1.08	2.82 ± 1.49
16	Ex-L	2.97 ± 1.69	3.17 ± 1.45
17	Pu-R	2.13 ± 1.01	1.51 ± 1.02
18	Pu-L	1.93 ± 0.92	1.28 ± 1.15
19	Zg-R	2.14 ± 0.85	2.19 ± 1.47
20	Zg-L	3.70 ± 1.63	3.05 ± 1.00
21	Ala-R	1.32 ± 0.68	1.03 ± 0.58
22	Ala-L	1.61 ± 0.67	1.04 ± 0.37
23	Sal-R	1.80 ± 0.85	1.36 ± 0.71
24	Sal-L	1.64 ± 0.75	1.10 ± 0.52
25	Tr-R	2.13 ± 0.74	1.34 ± 0.90
26	Tr-L	2.70 ± 0.92	1.67 ± 1.68
27	Go-R	5.10 ± 3.04	6.30 ± 3.09
28	Go-L	4.46 ± 2.31	5.53 ± 2.45
29	Cph-R	1.83 ± 0.84	1.33 ± 0.50
30	Cph-L	1.71 ± 1.08	0.96 ± 0.52
31	Ch-R	1.53 ± 0.59	1.03 ± 0.59
32	Ch-L	1.52 ± 0.68	0.95 ± 0.57
	Mean	2.34 ± 1.76	2.16 ± 1.97

**Table 3:** Landmark localization error for the 3D facial anatomical landmarks based on facial area (mm).

Area	TH Group ($\overset{-}{x}$ ± s)	MM Group ($\overset{-}{x}$ ± s)
Upper face (4)	4.10 ± 2.39	3.99 ± 2.47
Middle face (17)	1.95 ± 1.16	1.62 ± 1.22
Lower face (11)	2.31 ± 1.87	2.33 ± 2.27
Facial central area (26)	2.07 ± 1.43	1.81 ± 1.41
Facial marginal area (6)	3.52 ± 2.43	3.70 ± 3.04

**Table 4:** Results of related studies on the automatic determination of 3D facial anatomical landmarks.

Classification	Researcher/Year	Number of Landmarks	Mean Error (mm)
Geometric feature algorithms	Vezzetti E [16]/2012	9	3.86
	Liang S [18]/2013	10	3.12
	Liang Y [15]/2017	8	4.17
	Abu A [17]/2019	10	2.23
Artificial intelligence algorithms	Gilani SZ [21]/2017	11	3.00
	Wang K [22]/2018	14	3.96
	Wang L [19]/2018	22	3.71
	Paulsen RR [23]/2019	11	2.42
	Zhu Y [24]/2022	21	1.13
Template method	This paper, TH-OCR	32	2.34
	This paper, MeshMonk	32	2.16

## Data Availability

The datasets used and analyzed during the current study are available from the corresponding author on reasonable request.

## Abbreviations

3D: three-dimensional; 2D: two-dimensional; ICP: iterative closest point; PA: Procrustes analysis.

## References

[1] Hicks J.L. (2000). Important landmarks of the orofacial complex. Emerg. Med. Clin. N. Am..

[2] Kook M., Jung S., Park H., Oh H., Ryu S., Cho J., Lee J., Yoon S., Kim M., Shin H. (2013). A comparison study of different facial soft tissue analysis methods. J. Cranio-Maxillo-Facial Surg..

[3] Germec-Cakan D., Canter H.I., Nur B., Arun T. (2010). Comparison of Facial Soft Tissue Measurements on Three-Dimensional Images and Models Obtained with Different Methods. J. Craniofac. Surg..

[4] Ferrario V.F., Sforza C., Poggio C.E., Serrao G. (1996). Facial three-dimensional morphometry. Am. J. Orthod. Dentofac. Orthop..

[5] Dalal A.B., Phadke S.R. (2007). Morphometric analysis of face in dysmorphology. Comput. Meth. Prog. Bio..

[6] Storms A.S., Miclotte A., Grosjean L., Cadenas De Llano-Pérula M., Alqerban A., Fieuws S., Sun Y., Politis C., Verdonck A., Willems G. (2017). Short-term hard and soft tissue changes after mandibular advancement surgery in Class II patients: A retrospective cephalometric study. Eur. J. Orthodont..

[7] Salloum E., Millett D.T., Kelly N., McIntyre G.T., Cronin M.S. (2018). Soft tissue changes: A comparison between changes caused by the construction bite and by successful treatment with a modified Twin-block appliance. Eur. J. Orthodont..

[8] Ubaya T., Sherriff A., Ayoub A., Khambay B. (2012). Soft tissue morphology of the naso-maxillary complex following surgical correction of maxillary hypoplasia. Int. J. Oral. Maxillofac. Surg..

[9] Rosati R., De Menezes M., Da S.A., Rossetti A., Lanza A.G., Sforza C. (2014). Stereophotogrammetric evaluation of tooth-induced labial protrusion. J. Prosthodont..

[10] Allam E., Mpofu P., Ghoneima A., Tuceryan M., Kula K. (2018). The Relationship Between Hard Tissue and Soft Tissue Dimensions of the Nose in Children: A 3D Cone Beam Computed Tomography Study. J. Forensic. Sci..

[11] Zhu Y., Fu X., Zhang L., Zheng S., Wen A., Xiao N., Wang Y., Zhao Y. (2022). A mathematical algorithm of the facial symmetry plane: Application to mandibular deformity 3D facial data. J. Anat..

[12] Farkas L.G., Bryson W., Klotz J. (1980). Is photogrammetry of the face reliable?. Plast. Reconstr. Surg..

[13] Ma L., Xu T., Lin J. (2009). Validation of a three-dimensional facial scanning system based on structured light techniques. Comput. Meth. Prog. Biomed..

[14] Sun H., Bi D., Tao J. (2009). Markers localization of 3D face based on curvature and geometric constraints. Microcomput. Inf..

[15] Liang Y., Zhang Y. (2017). 3D facial landmark localization under pose and expression variations. Control. Theory Appl..

[16] Vezzetti E., Moos S., Marcolin F., Stola V. (2012). A pose-independent method for 3D face landmark formalization. Comput. Meth. Prog. Biomed..

[17] Abu A., Ngo C.G., Abu-Hassan N., Othman S.A. (2019). Automated craniofacial landmarks detection on 3D image using geometry characteristics information. BMC Bioinform..

[18] Liang S., Wu J., Weinberg S.M., Shapiro L.G. (2013). Improved detection of landmarks on 3D human face data. Annu. Int. Conf. IEEE Eng. Med. Biol. Soc..

[19] Wang L. (2019). Research of Robust 3d Facial Landmarking Techniques.

[20] Cheng X., Da F., Deng X. (2018). Coarse-to-fine 3D facial landmark localization based on keypoints. Chin. J. Sci. Instrum..

[21] Gilani S.Z., Mian A., Eastwood P. (2017). Deep, dense and accurate 3D face correspondence for generating population specific deformable models. Pattern Recogn..

[22] Wang K., Zhao X., Gao W., Zou J. (2018). A coarse-to-fine approach for 3D facial landmarking by using deep feature fusion. Symmetry.

[23] Paulsen R.R., Juhl K.A., Haspang T.M., Hansen T., Ganz M., Einarsson G. (2019). Multi-view Consensus CNN for 3D Facial Landmark Placement. Proceedings of the Computer Vision–ACCV 2018: 14th Asian Conference on Computer Vision.

[24] Zhu Y., Xu Q., Zhao Y., Zhang L., Fu Z., Wen A., Gao Z., Zhang Y., Fu X., Wang Y. (2022). Deep learning-assisted construction of three-demensional facial midsagittal plane. J. Peking Univ. (Health Sci.).

[25] White J.D., Ortega-Castrillón A., Matthews H., Zaidi A.A., Ekrami O., Snyders J., Fan Y., Penington T., Van Dongen S., Shriver M.D. (2019). MeshMonk: Open-source large-scale intensive 3D phenotyping. Sci. Rep..

[26] Wen A., Zhu Y., Zheng S., Xiao N., Gao Z., Fu X., Wang Y., Zhao Y. (2022). Preliminary study on the method of automatically determining facial landmarks based on three-dimensional face template. Chin. J. Stomatol..

[27] Li M., Cole J.B., Manyama M., Larson J.R., Liberton D.K., Riccardi S.L., Ferrara T.M., Santorico S.A., Bannister J.J., Forkert N.D. (2017). Rapid automated landmarking for morphometric analysis of three-dimensional facial scans. J. Anat..

[28] Gilani S.Z., Shafait F., Mian A. Shape-based automatic detection of a large number of 3D facial landmarks. Proceedings of the IEEE Conference on Computer Vision and Pattern Recognition.

[29] Li Z., Giunta R.E., Frank K., Schenck T.L., Koban K.C. (2022). Reproducibility of Novel Soft-Tissue Landmarks on Three-Dimensional Human Facial Scan Images in Caucasian and Asian. Aesthet. Plast. Surg..

[30] Baysal A., Sahan A.O., Ozturk M.A., Uysal T. (2016). Reproducibility and reliability of three-dimensional soft tissue landmark identification using three-dimensional stereophotogrammetry. Angle Orthod..

[31] Kung S., Walters M., Claes P., Goldblatt J., Le Souef P., Baynam G. (2013). A Dysmorphometric Analysis to Investigate Facial Phenotypic Signatures as a Foundation for Non-invasive Monitoring of Lysosomal Storage Disorders. Jimd. Rep..

[32] Farnell D.J.J., Richmond S., Galloway J., Zhurov A.I., Pirttiniemi P., Heikkinen T., Harila V., Matthews H., Claes P. (2021). An exploration of adolescent facial shape changes with age via multilevel partial least squares regression. Comput. Meth. Prog. Bio.

[33] Ekrami O., Claes P., White J.D., Zaidi A.A., Shriver M.D., Van Dongen S. (2018). Measuring asymmetry from high-density 3D surface scans: An application to human faces. PLoS ONE.

[34] Claes P., Walters M., Vandermeulen D., Clement J.G. (2011). Spatially-dense 3D facial asymmetry assessment in both typical and disordered growth. J. Anat..

[35] Fagertun J., Harder S., Rosengren A., Moeller C., Werge T., Paulsen R.R., Hansen T.F. (2014). 3D facial landmarks: Inter-operator variability of manual annotation. BMC Med. Imaging.

